# Association of Vaccine Type and Prior SARS-CoV-2 Infection With Symptoms and Antibody Measurements Following Vaccination Among Health Care Workers

**DOI:** 10.1001/jamainternmed.2021.4580

**Published:** 2021-08-16

**Authors:** Amanda K. Debes, Shaoming Xiao, Elizabeth Colantuoni, Emily R. Egbert, Patrizio Caturegli, Avinash Gadala, Aaron M. Milstone

**Affiliations:** 1 Johns Hopkins School of Public Health, Baltimore, Maryland; 2 Johns Hopkins University School of Medicine, Baltimore, Maryland; 3 Johns Hopkins Health System, Baltimore, Maryland

## Abstract

This cohort study evaluates symptoms following vaccination and antibody measurements in hospital workers who received an mRNA SARS-CoV-2 vaccine and had prior SARS-CoV-2 infection.

Two messenger RNA (mRNA) vaccines (Pfizer-BioNTech and Moderna) encoding the spike protein of SARS-CoV-2 induce the production of spike antibodies that neutralize SARS-CoV-2^1^ and are clinically effective against COVID-19.^2^ These vaccines can elicit greater local and systemic reactions in persons with prior SARS-CoV-2 infection.^3^ Whether symptoms following vaccination are associated with effectiveness is unknown, and, therefore, anxiety can arise in persons who did not develop a reaction following vaccination.^4^ We evaluated symptoms following vaccination and serum spike antibody levels in a cohort of hospital workers (HWs) who received either mRNA vaccine and had known status of prior SARS-CoV-2 infection to identify differences in symptoms and serum immunoglobulin G (IgG) antibodies against S1 spike protein.

## Methods

In June 2020, HWs in the Johns Hopkins Health System provided oral informed consent to participate in a longitudinal study of S1 spike antibodies in which serum samples and survey responses were collected every 3 to 4 months. Ethical approval was obtained from the Johns Hopkins University Institutional Review Board. The HWs who participated for a study visit between March 10 and April 8, 2021, were included in this analysis if their serum sample was collected 14 or more days after receiving dose 2 of either mRNA vaccine. Using an enzyme-linked immunosorbent assay (Euroimmun), IgG antibody measurements were determined based on optical density ratios with an upper threshold of 11 based on assay saturation.^1^ Prior SARS-CoV-2 infection was defined as having (1) a positive SARS-CoV-2 polymerase chain reaction test result prior to 14 days after dose 2 or (2) S1 spike IgG measurement greater than 1.23 prior to vaccination.^5^ Participants self-reported symptoms following vaccination as none, mild (injection site pain, mild fatigue, headache), or clinically significant (fatigue, fever, chills). Logistic regression models were used to explore the association of prior SARS-CoV-2 infection and vaccine type with symptoms following each dose, adjusting for sex and age. A linear regression model was used to explore the association between magnitude of antibody response (log-transformed) and age, sex, prior infection, vaccine type, symptoms, and time after 2 doses of vaccine. Analyses were performed in R, version 4.0.2 (R Foundation).

## Results

A questionnaire and serum sample were collected 14 or more days following dose 2 for 954 HWs. Clinically significant symptoms were reported by 52 of the 954 (5%) after dose 1 and 407 (43%) after dose 2. After adjusting for prior SARS-CoV-2 infection, age, and sex, the odds of clinically significant symptoms following either dose were higher among participants who received the Moderna vs the Pfizer vaccine (dose 1: odds ratio [OR], 1.83; 95% CI, 0.96-3.50; dose 2: OR, 2.43; 95% CI, 1.73-3.40) (Table). Prior SARS-CoV-2 exposure was associated with increased odds of clinically significant symptoms following dose 1 (OR, 4.38; 95% CI, 2.25-8.55) but not dose 2 (OR, 0.60; 95% CI, 0.36-0.99), after controlling for vaccine type, age, and sex.

**Table.** Significant Symptoms and Antibody Measurement Following SARS-CoV-2 mRNA Vaccines

Characteristic	Significant symptoms		
	Following dose 1	Following dose 2	Following dose 1 or 2
**Adjusted odds ratio (95% CI) of symptoms following dose 1, dose 2, either dose**			
Significant symptoms following dose 1	NA	1.21 (0.67-2.17)	NA
Age >60 y	1.42 (0.64-3.14)	0.46 (0.29-0.72)	0.47 (0.31-0.73)
Male sex^a^	0.82 (0.37-1.79)	0.88 (0.63-1.25)	0.88 (0.63-1.24)
Vaccine type^b^: Moderna	1.65 (0.87-3.11)	2.44 (1.75-3.42)	2.33 (1.67-3.26)
Prior SARS-CoV-2 infection	4.59 (2.36-8.92)	0.60 (0.36-0.99)	0.83 (0.51-1.33)
**Median antibody measurement (IQR) and adjusted relative median antibody measurement (95% CI) >14 d following second dose vaccine**			
	**Median antibody measurement of each group**		**Relative median antibody measurement** ^c^
	**Yes**	**No**	
Significant symptoms	8.82 (8.04-9.68)	8.46 (7.62-9.16)	1.05 (1.03-1.07)
Age >60 y	8.39 (7.26-9.16)	8.62 (7.89-9.43)	0.92 (0.88-0.96)
Male sex	8.41 (7.65-9.11)	8.66 (7.85-9.48)	0.95 (0.92-0.98)
Vaccine type: Moderna	9.28 (8.45-10.59)	8.51 (7.70-9.22)	1.09 (1.06-1.11)
Prior SARS-CoV-2 infection	9.28 (8.56-11.00)	8.56 (7.80-9.33)	1.10 (1.07-1.14)

Abbreviations: IQR, interquartile range; NA, not applicable.

^a^ Reference group: Female; 3 participants reported other sex, all of whom reported mild or no symptoms after dose 1, and 1 of them reported significant symptoms after dose 2. The antibody measurements for them were 6.53, 8.98, and 8.16 separately.

^b^ Reference group: Pfizer.

^c^ Time since 14 days after dose 2 and other covariates have been adjusted. The 95% CIs were constructed via the percentile bootstrap procedure using 10 000 bootstrap samples.

Regardless of symptoms, the vast majority of participants (953 of 954, greater than 99.9%) developed spike IgG antibodies 14 or more days following dose 2; 1 participant who was taking immunosuppressant medication did not develop IgG antibodies (Figure). Reporting clinically significant symptoms, age younger than 60 years, female sex, receipt of Moderna vaccine, and prior SARS-CoV-2 exposure were independently associated with higher median IgG measurements, after adjusting for time after dose 2.

**Figure.**
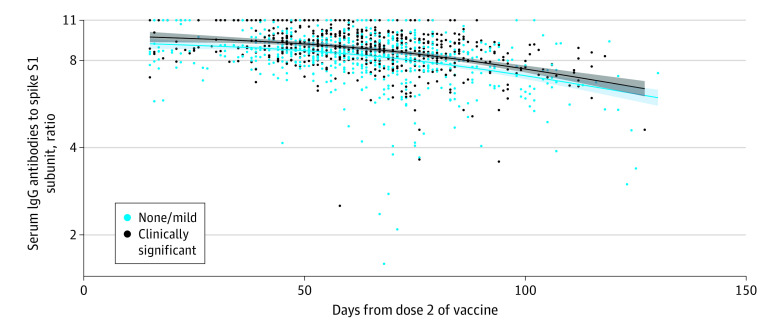
Antibody Measurement More Than 14 Days Following Dose 2 of SARS-CoV-2 Messenger RNA (mRNA) Vaccines Over Time Stratified by Symptoms Relationship of serum immunoglobulin G (IgG) antibodies recognizing the S1 subunit of spike and days after the second dose of SARS-CoV-2 mRNA vaccine in 953 hospital workers (1 participant who was receiving immunosuppressant medication did not develop IgG antibodies and is not shown). The IgG antibody measurements represent the ratio of 2 optical densities (ODs): the OD of the patient serum over the OD of an assay calibrator provided by the manufacturer. A measurement greater than 1.23 indicates the presence of spike antibodies with an upper threshold of 11 based on assay saturation.^1,5^ Antibody measurements were stratified by symptoms after either vaccine dose. The curves were predicted median antibody measurement over time (time was allowed as a natural cubic spline with 2 degrees of freedom) stratified by symptoms, set age, sex, vaccine type, and prior infection as the sample average. Shaded areas represent 95% CIs.

## Discussion

Nearly 100% of HWs in this study mounted a strong antibody response to the spike protein after dose 2 of the SARS-CoV-2 mRNA vaccine independent of vaccine-induced reactions. Clinically significant symptoms following dose 1 were associated with prior SARS-CoV-2 infection, confirming prior reports.^4^ Clinically significant symptoms following vaccination were more frequent following dose 2 and receipt of the Moderna vaccine.^3^

This study included participants within a longitudinal cohort study, leading to 2 potential limitations. First, the timing of survey collection may have led to recall bias and affected symptom reporting. Second, immune response was measured by enzyme-linked immunosorbent assay and not neutralizing antibody titers.

Spike IgG antibody measurements were higher in HWs who received the Moderna vaccine, had prior SARS-CoV-2 infection, and reported clinically significant reactions. The role of higher antibody levels in preventing COVID-19 and providing lasting immunity remains unknown, however. Overall, the findings suggest that regardless of vaccine reactions or prior SARS-CoV-2 infection, either spike mRNA vaccine will provide a robust spike antibody response.
